# The Painful Anterior Apprehension Test – an Indication of Occult Shoulder Instability

**DOI:** 10.5704/MOJ.2203.014

**Published:** 2022-03

**Authors:** GW Law, ZD Ng, JH Tan, KLF Wong, YH Ng

**Affiliations:** 1 Department of Orthopaedic Surgery, National University Health System, Singapore; 2 Department of Orthopaedic Surgery, Sengkang General Hospital, Singapore

**Keywords:** painful apprehension test, shoulder instability, synovitis, dislocation

## Abstract

**Introduction:**

To evaluate the clinical relevance of the painful anterior apprehension test in shoulder instability.

**Materials and methods:**

We performed a retrospective study of 155 patients that underwent arthroscopic anterior Bankart repair between 2014–2016. Exclusion criteria were previous ipsilateral shoulder surgery, bony Bankart lesions, glenohumeral osteoarthritis and concomitant surgery involving rotator cuff tears, biceps tendon pathology and superior labrum from anterior to posterior (SLAP) lesions. The study cohort was divided into three groups: apprehension test with apprehension only, apprehension test with pain only, and apprehension test with both apprehension and pain. Patient demographics, clinical characteristics, radiological imaging, arthroscopy findings and surgical outcomes (Constant, American Shoulder and Elbow Surgeons (ASES), SF-36 scores) were evaluated.

**Results:**

A total of 115 (74.2%) had apprehension only, 26 (16.8%) had pain only and 14 (9.0%) had pain and apprehension with the apprehension test. Univariate analysis showed significant differences between the groups in patients with traumatic shoulder dislocation (p=0.028), patients presenting with pain (p=0.014) and patients presenting with recurrent dislocations (p=0.046). Patients with a purely painful apprehension test were more likely to have a traumatic shoulder dislocation, more likely to present only with pain, and less likely to present with recurrent shoulder dislocations. Multivariate analysis showed that none of these factors alone were significant as single predictors for shoulder instability. All three groups were otherwise similar in patient profile, MRI and arthroscopic assessments, and clinical outcomes of surgery. Excellent clinical outcomes were achieved in all groups with no difference in pre-operative and post-operative scores across all groups at all time points.

**Conclusion:**

The painful apprehension test may suggest underlying shoulder instability.

## Introduction

The anterior apprehension test is routinely used to detect anterior shoulder instability with several studies showing the diagnostic value of this simple clinical test^1,2^. A level 1 prospective cohort study showed that the overall accuracy of the apprehension test was 81.7%^2^, while a separate meta-analysis also showed a strong association between a positive apprehension test and anterior shoulder instability with a high positive likelihood ratio^3^. In most of these previous studies the hallmark of a positive apprehension test was apprehension or a sense of instability rather than pain.

The painful apprehension test had been previously described as a possible indication of an underlying subtle anterior shoulder instability in the overhand or throwing athlete presenting with pain^4-6^. The unstable painful shoulder (UPS) and multidirectional instability (MDI) are two forms of shoulder instability that can present with pain that are often missed^6^. UPS can occur without an apparent history of glenohumeral dislocation or subluxation^7^ and multidirectional instability (MDI) often do not have a history of trauma^6^, making instability as a cause of shoulder pain particularly difficult to diagnose.

Farber *et al*^1^ reported that the specificity and overall accuracy of the anterior apprehension test decreased significantly when pain rather than apprehension was the diagnostic criteria used in the clinical test. The co-existence of a variety of other pathologies such as a partial rotator cuff tear, rotator cuff tendinitis, biceps tendinitis, posterior labral tear or superior labral anterior posterior (SLAP) tear can similarly cause a painful apprehension test which may be a cause for the reduced specificity of this clinical test^8^. A dedicated study on this subject of patients with a painful apprehension test has never been carried out to the authors’ knowledge.

The objectives of this study were to (i) evaluate the clinical relevance of the painful anterior apprehension test, (ii) correlate the painful apprehension test with MRI and arthroscopic findings and (iii) understand the underlying patho-mechanism of the painful apprehension test.

## Materials and Methods

This is a retrospective review of all patients that underwent arthroscopic anterior Bankart repair between 2014-2016 at a tertiary hospital. All surgeries were performed by two fellowship-trained shoulder surgeons during the study timeframe. Ethical approval by the hospital’s ethics committee was secured for this study.

All patients with surgical stabilisation of an anterior Bankart lesion confirmed on arthroscopy were included. Patients with concomitant surgery involving rotator cuff tears, biceps tendon pathology and SLAP lesions were excluded as these were confounding factors with pain generators addressed in the same setting affecting outcome correlation. Patients with bony Bankart lesions, glenohumeral osteoarthritis and previous ipsilateral shoulder surgery were also excluded as they were potential pain generators that could confound results of the study.

All patients were interviewed and baseline demographic data collected. These included age, gender, arm dominance, smoking history, sporting activity and occupation. Presenting complaints of pain, number of dislocations and severity of the trauma incurred at the first dislocation were recorded.

Besides a general shoulder examination, the Beighton score for joint laxity was also recorded. Patients with Beighton score ≥4 were deemed to have generalised joint laxity or hypermobility. The anterior apprehension test was then conducted and apprehension or pain during the manoeuvre was recorded. The study cohort was divided into three groups according to the test results: (i) apprehension test with apprehension only, (ii) apprehension test with pain only, and (iii) apprehension test with both apprehension and pain. The groups were then compared.

All patients had pre-operative plain radiographs with anterior/posterior and axillary views as well as an MRI of the affected shoulder. The presence of Bankart lesions, Hill-Sach’s lesions, SLAP tears, biceps tendon lesions and rotator cuff pathology were recorded. Pre-operative Constant, ASES and SF-36 scores were documented for all patients.

All patients underwent arthroscopic assessment of the shoulder with the patient in the beach chair position. An arthroscopic Bankart repair was carried out using standard portals. A mean of 4 (range 3-5) suture anchors were used.

Post-operatively the arm was immobilised for three weeks in an arm sling. Pendulum exercises were commenced at three weeks, followed by a standardised physiotherapy protocol involving passive followed by active range of motion exercises. External rotation beyond neutral was not allowed until six weeks after surgery. Strengthening exercises were commenced at eight weeks post-surgery.

Post-operative Constant, ASES and SF-36 scores were documented at 6 months, 12 months, and 24 months after surgery.

Data was compiled and analysed using SPSS version 21 [SPSS Inc., Chicago, Illinois, USA]. The Chi-Square test was used for analysis of categorical variables. Continuous variables were analysed for normality using the Shapiro-Wilk test; normally distributed variables were analysed using parametric tests, otherwise non-parametric tests were used. One-way ANOVA and Tukey post hoc tests were used for univariate analysis of continuous variables. A multinomial logistics regression model was used to identify risk factors for shoulder instability in those with the painful apprehension test. Only significant covariates after univariate analysis are included in the multivariate modelling. Statistical significance was set at p <0.05.

## Results

There were no significant differences between all 3 groups in term of gender, mean age at presentation, handedness, smoking history, involvement in high risk occupations, participation in competitive sports or symptom duration, and severity of trauma incurred at the first dislocation (Table I).

**Table I:** Patient profile, demographics and clinical characteristics

	Anterior Apprehension Test Results			p-value
	Apprehension only (n=115)	Pain only	Pain and apprehension (n=26) (n=14)	
Gender (%)				
Male	112 (97.4)	14 (100.0)	14 (100.0)	0.076
Female	3 (2.6)	3 (11.5)	0 (0.0)	
Age (Median, Range) Handedness (%)	25.3 ± 9.7	25.7 ± 2.8	20.1 ± 6.9	0.123
Right	98 (85.2)	24 (92.3)	13 (92.9)	0.501
Left	17 (14.8)	2 (7.7)	1 (7.1)	
Smoker (%)				
No	93 (80.9)	24 (92.3)	13 (92.9)	0.860
Yes	22 (19.1)	2 (7.7)	1 (7.1)	
Traumatic Dislocation (%)				
Yes	80 (69.6)	21 (80.8)	3 (21.4)	**0.028**
No	35 (30.4)	5 (19.2)	11 (78.6)	
Presenting Complaint: Pain (%)				
Yes	35 (30.4)	16 (61.5)	6 (42.9)	**0.014**
No	80 (69.6)	10 (38.5)	8 (57.1)	
Presenting Complaint: Recurrent Dislocation (%)				
Yes	55 (47.8)	6 (23.1)	4 (28.6)	**0.046**
No	60 (52.2)	20 (76.9)	10 (71.4)	
	19.2±17.1	15.9±13.9	17.2±16.4	0.623
Symptoms Duration (months) (Mean±SD) Competitive Sports (%)				
No	40 (34.8)	9 (34.6)	7 (50.0)	0.532
Yes	75 (65.2)	17 (65.4)	7 (50.0)	
High Risk Occupation (%)				
No	53 (46.1)	12 (46.2)	4 (28.6)	0.458
Yes	62 (53.9)	14 (53.8)	10 (71.4)	

SD = Standard deviation

Bold indicates statistical significance

Univariate analysis showed significant differences between the 3 groups in patients with a history of traumatic shoulder dislocation (p=0.028), patients with pain as the presenting complaint (p=0.014) and patients with recurrent dislocations as the presenting complaint (p=0.046) (Table I). Patients with the painful apprehension test were more likely to have a history of traumatic dislocation, more likely to present with pain, and less likely to present with recurrent shoulder dislocations (Table I). However, none of these factors were shown to be significant as single predictors for shoulder instability on multivariate analysis (Table II).

**Table II:** Multinomial logistics regression analysis to identify risk factors for shoulder instability in patients with the painful apprehension test

Parameter	Multivariate Analysis	
	p-value	OR
Traumatic Dislocation	0.973	0.181 –5.227
Presenting Complaint: Pain	0.191	0.07 –1.99
Presenting Complaint: Recurrent Dislocation	0.525	0.102 –4.354

Similar rates of detecting Bankart lesions, Hill-Sachs lesions, rotator cuff pathology and SLAP lesions were found across all groups for both MRI and arthroscopic assessments. (Table III). On clinical examination except for the findings of the apprehension test, no other differences were noted, including the severity of joint laxity.

**Table III:** MRI and intra-operative arthroscopy findings

	Anterior Apprehension Test Results			p-value
	Apprehension only (n=115)	Pain only (n=26)	Pain and apprehension (n=14)	
Bankart (MRI), no. (%)				
Yes	92 (80.0)	19 (73.15)	10 (71.4)	0.615
No	23 (20.0)	7 (26.9)	11 (28.6)	
Hill Sachs (MRI), no. (%)				
Yes	72 (62.6)	11 (42.3)	6 (42.9)	0.087
No	43 (37.4)	15 (57.7)	8 (57.1)	
Hill Sachs (Intra-op), no. (%)				
Yes	57 (49.6)	8 (30.8)	4 (28.6)	0.100
No	58 (50.4)	18 (69.2)	10 (71.4)	
SLAP (MRI), no. (%)				
Yes	11 (9.6)	4 (15.4)	1 (7.1)	0.629
No	104 (90.4)	22 (84.6)	13 (92.9)	
SLAP (Intra-op), no. (%)				
Yes	2 (1.7)	2 (7.7)	0 (0.0)	0.186
No	113 (98.3)	24 (92.3)	14 (100.0)	
RC Pathology (MRI), no. (%)				
Yes	11 (9.6)	2 (7.7)	0 (0.0)	0.476
No	104 (90.4)	24 (92.3)	14 (100.0)	
RC Pathology (Intra-op), no. (%)				
Yes	3 (2.6)	0 (0.0)	0 (0.0)	0.592
No	112 (97.4)	26 (100.0)	14 (100.0)	
Hyperlaxity (Intra-op), no. (%)				
Yes	14 (12.2)	5 (19.2)	3 (21.4)	0.471
No	101 (87.8)	21 (80.8)	11 (78.6)	

RC = Rotator cuff

Bold indicates statistical significance

Anterior labral repair was performed in all patients across all three groups. One of the 26 patients in the group with pain only with the apprehension test had an additional posterior lateral repair, while 2 of the 115 patients in the group with apprehension only had a posterior labral repair. There were no additional other surgical procedures performed for all other patients.

All 3 groups showed significant improvement in the Constant, ASES and SF-36 scores at an average of 16 months post-surgery where this data was available with no significant difference in scores at all time points pre-and post-operatively.

## Discussion

Our study showed that the painful apprehension test occurred in 25.8% of patients (40/155 patients) with Bankart lesions that underwent arthroscopic repair, of which two-thirds (26/40 patients) were in the purely painful form. Importantly, arthroscopic stabilisation achieved excellent clinical outcomes in this subgroup of patients with the painful apprehension test, with significant improvements in the Constant, ASES and SF-36 scores, similar to patients with apprehension alone in the treatment of shoulder instability.

The anterior apprehension test of the shoulder performed with the shoulder in 90o of abduction and maximal external rotation, with the elbow in 90o of flexion has traditionally been used to clinically diagnosis anterior shoulder instability with great reliability^2,3,9^. Several authors have also reported that apprehension, rather than pain as the key criterion for the accurate diagnosis of instability using this clinical test^1,10,11^.

Farber *et al* reported 93% overall accuracy of the anterior apprehension test when apprehension was used as the positive test criterion, which dropped to 55% when pain was used as the positive test criterion^1^. However, a closer look at the diagnostic value of an apprehensive apprehension test would reveal that despite the excellent specificity of the test of 96%, its sensitivity in picking up positive cases is only 72% in that study. We postulate that a proportion of these “missed” cases might belong to the group with a purely painful apprehension test. While it may be true that doing MRI for all patients with a painful apprehension would lead to a high rate of negative scans, completely ignoring the purely painful apprehension test would lead to a significant rate of missed lesions that would have been managed appropriately with surgery. In our study of Bankart lesions managed with arthroscopic stabilisation where satisfactory outcomes were achieved, 16.8% of patients demonstrated apprehension tests in the purely painful form, involving patients with and without a prior history of traumatic shoulder dislocation.

The patient profile of the group with the purely painful apprehension test was similar to those with apprehension in terms of age, gender distribution, hand dominance and involvement in high risk occupations or competitive sports, with the majority having a history of prior traumatic dislocation (80.8%). With the majority of our patients with the purely painful apprehension test presenting with shoulder pain as a significant symptom at their first clinical visit (61.5% versus 30.4%) and lower rates of recurrent dislocation (23.1%% versus 47.8%) compared to patients with a purely apprehensive apprehension test, surgeons should have a high clinical suspicion for shoulder instability as the cause of the pain in the presence of a Bankart lesion especially if other findings on imaging were deemed insignificant or asymptomatic with bedside tests. In this group of patients, pain was likely the protective mechanism limiting their shoulder range of motion before the shoulder could exceed its arc of stability. As such, rather than instability, they would more likely present with persistent shoulder pain and activity limitation instead. The abduction, external rotation (ABER) manoeuvre translates the humeral head antero-inferiorly causing it to “roll over” the Bankart lesion, which may not be extensive enough to destabilise the shoulder but adequately disrupted to cause pain. The universal arthroscopic finding in our series in patients with a painful shoulder was focal capsular synovitis around the frayed torn edges of the labrum, which was the likely pain generator. Repetitive ABER motion might potentially perpetuate this “roll-over synovitis” leading to the predominant symptom of pain which may distract patients from a sensation of instability. We found no significant difference in the incidence of Bankart lesions, cuff pathology or SLAP lesions between patients with and without pain on the apprehension test, making these other pathologies less likely causes of pain. Interestingly, the rates of Hill-Sachs lesions found in patients with apprehension only (49.6%) was higher than patients with who had pain (30.8%). This provided circumstantial evidence supporting our postulation that pain during the ABER manoeuvre might be protective against frank shoulder dislocations resulting in impaction fractures of the humeral head.

Furthermore, the proportion of patients with a preceding traumatic dislocating event in the group with a painful apprehension test was higher than the group with a purely apprehensive test (80.8% vs 69.9%), and the latter group was also nearly twice as likely to have ligamentous laxity than the former (14.8% vs 7.7%). This suggested a greater role of trauma, rather than ligamentous laxity, as the underlying aetiology for patients with a painful “roll-over” lesion, with predominant symptoms of pain rather than instability. These associations were not statistically significantly, and this is likely due to the underpowered nature of our study but they were certainly clinically relevant.

Boileau *et al* emphasised the importance of recognising UPS early in the young, hyperlax athlete complaining of deep, anterior shoulder pain as soft tissue or bony lesions indicative of instability may be found in the absence of an apparent history of shoulder subluxation or dislocation which were amenable to surgery with excellent outcomes^7^. Twenty patients were identified over a 5-year period with data collected prospectively and followed-up for a minimum of 2 years (mean 38 months, range 24-76 months). Despite the small numbers, the painful apprehension test was reproducible in all 20 patients (100%) involved, with 3 patients (15%) demonstrating both pain and apprehension with the test. A total of 85% of patients in this group demonstrated purely painful apprehension tests. Apprehension alone was not elicited. Future studies to identify specific circumstances or in subgroups of patients with unique characteristics may be useful in increasing the specificity of this test for shoulder instability.

Numerous clinical tests have been developed to assess anterior shoulder instability. In van Kampen *et al’s* study of six of these clinical tests (apprehension, relocation, release, anterior drawer, load and shift, and hyperabduction tests) for traumatic anterior shoulder instability, the relocation test was the most sensitive (96.7%), the anterior drawer test was the most specific (92.7%), and the release test showed the best overall accuracy (86.4%)^2^, all of which were dependent on whether apprehension or pain was reproduced or relieved with the manoeuvre. Lafosse *et al* assessed instability by comparing of the range of motion in the abnormal shoulder with the normal side using the hyper extension-internal rotation (HERI) test^12^. Isolated inferior gleno-humeral ligament (IGHL) section in the cadaveric arm of the study produced a mean increase of 14.5° in gleno-humeral extension, similar to their clinical arm of 50 patients with chronic unilateral anterior gleno-humeral instability when comparing the normal and abnormal sides^12^. Although this test does not induce apprehension most of the time, allowing accurate measurements of the extension angles and assessments of gleno-humeral laxity, this test requires a normal contralateral shoulder for side-to-side comparison as a prerequisite. With no single clinical test being both highly sensitive and specific to date, using multiple clinical tests combined may be required to avoid underdiagnosing subtle shoulder instability and delayed treatment.

Instability of the shoulder can exist in the purely painful form, with or without a history of traumatic shoulder dislocation, recurrent dislocation or ligamentous laxity. Our study shows that the painful apprehension test is common in shoulder instability, particularly in patients presenting with pain as their main symptom despite a history of traumatic shoulder dislocation. These patients tend to have lower rates of recurrent dislocation but can achieve predictable, satisfactory outcomes with arthroscopic stabilisation of their Bankart lesions.

The strengths of our study are: (i) having 2 fellowship-trained shoulder surgeons who work closely with each other ensures uniformity in clinical assessments, patient selection and quality of surgeries performed, thereby reducing the number of variables from poor standardisation, and (ii) utilisation of multiple outcome measures provides a more holistic assessment. Our study limitations include: (i) the retrospective design of the study limiting collection of more comprehensive data, and (ii) relatively small sample size.

## Conclusion

The painful apprehension test should not be dismissed as a negative finding for shoulder instability. The underlying pain generator in this group of patients is likely the focal capsular synovitis around the torn labrum and persistent “rolling over” of the humeral head over the torn labrum perpetuates the painful synovitis. These patients are likely to present with shoulder pain and fewer episodes of dislocation. Clinicians should have a lower threshold for advanced imaging to identify these patients early.
